# The distinct clinical trajectory, metastatic sites, and immunobiology of microsatellite-instability-high cancers

**DOI:** 10.3389/fgene.2022.933475

**Published:** 2022-12-01

**Authors:** Shuting Han, Aik Yong Chok, Daniel Yang Yao Peh, Joshua Zhi-Ming Ho, Emile Kwong Wei Tan, Si-Lin Koo, Iain Bee-Huat Tan, Johnny Chin-Ann Ong

**Affiliations:** ^1^ Division of Medical Oncology, National Cancer Centre Singapore, Singapore, Singapore; ^2^ Department of Colorectal Surgery, Singapore General Hospital, Singapore, Singapore; ^3^ Department of Sarcoma, Peritoneal and Rare Tumors (SPRinT), Division of Surgery and Surgical Oncology, National Cancer Centre Singapore, Singapore, Singapore

**Keywords:** microsatellite, instability, cancer, colorectal cancer, immunobiology, mismatch repair (MMR), therapy, Lynch Syndrome

## Abstract

Microsatellite-instability-high (MSI-H) cancers form a spectrum of solid organ tumors collectively known as Lynch Syndrome cancers, occurring not only in a subset of colorectal, endometrial, small bowel, gastric, pancreatic, and biliary tract cancers but also in prostate, breast, bladder, and thyroid cancers. Patients with Lynch Syndrome harbor germline mutations in mismatch repair genes, with a high degree of genomic instability, leading to somatic hypermutations and, therefore, oncogenesis and cancer progression. MSI-H cancers have unique clinicopathological characteristics compared to their microsatellite-stable (MSS) counterparts, marked by a higher neoantigen load, immune cell infiltration, and a marked clinical response to immune checkpoint blockade. Patients with known Lynch Syndrome may be detected early through surveillance, but some patients present with disseminated metastatic disease. The treatment landscape of MSI-H cancers, especially colorectal cancers, has undergone a paradigm shift and remains to be defined, with immune checkpoint blockade coming to the forefront of treatment strategies in the stage IV setting. We summarize in this review the clinical features of MSI-H cancers with a specific interest in the pattern of spread or recurrence, disease trajectory, and treatment strategies. We also summarize the tumor-immune landscape and genomic profile of MSI-H cancers and potential novel therapeutic strategies.

## Background

Microsatellite-instability-high (MSI-H) cancers are a unique class of tumors that may arise from somatic inactivating mutations or epigenetic silencing of mismatch repair (MMR) pathway genes, namely, mutL homologue 1 (MLH1), mutS homologue 2 (MSH2), mutS homologue 6 (MSH6), and postmeiotic segregation increased 2 (PMS2), or from germline mutations in the mismatch repair pathway genes. They are clinically distinct from their microsatellite-stable (MSS) counterparts and have become increasingly studied in view of their genomic instability, high somatic mutational burden, favorable immunobiology, and relatively indolent clinical trajectory.

Proteins within the MMR pathway include not only MLH1, MSH2, MSH6, and PMS2 proteins but also mutL homologue 3 (MLH3), human mutS homologue 3 (MSH3), postmeiotic segregation increased 1 (PMS1) proteins, and exonuclease 1 (Exo1). DNA mismatch errors are detected and bound by MSH2/MSH6 and MSH2/MSH3 (less commonly) heterodimers. MLH1/PMS2 heterodimers are then recruited for excision and synthesis of a corrected DNA strand. However, defective MMR genes or epigenetic silencing results in MMR protein loss and can, therefore, lead to loss of function of the aforementioned heterodimers and MMR complex. Deficient MMR (dMMR) leads to a strong mutator phenotype known as MSI-H, which is marked by polymorphisms in various microsatellite loci and high somatic mutations characterized by mainly frameshift mutations (Fishel, 2015). Other mutations that can lead to the MSI-H/dMMR phenotype include epithelial cellular adhesion molecule (EPCAM) deletions (leading to epigenetic inactivation of MSH2). Moreover, mutations in the exonuclease domain of DNA polymerase ɛ (POLE) can result in a hypermutated phenotype with even higher mutational burden than the dMMR phenotype, but they are not discussed in this study.

MSI-high tumors represent a constellation of cancers that range from endometrial, colorectal, gastric, biliary tract, pancreatic, bladder, thyroid, breast, prostate, and ovarian cancers to leukemia and central nervous system (CNS) tumors. The term “Hereditary Non-Polyposis Colorectal Cancer” or HNPCC was interestingly first used by Lynch in 1985, which was preceded by decades of observation of familial predominance of early onset colon cancers. The subsequent discovery of the genetic basis of this condition led the term Lynch Syndrome (LS) to refer to patients with autosomal dominant germline-deficient MMR genes. Around 15% of early-stage colorectal cancers, around 5% in advanced stage colorectal cancers, and 20%–30% of endometrial cancers are MSI-H—overall accounting for a significant clinical burden of disease. Amongst MSI-H colorectal cancers, a majority arose from sporadic etiology (MLH1 promoter silencing) while the remaining arose from germline Lynch Syndrome. LS is a clinically significant hereditary cancer syndrome—an estimated 1 in 300 people in the United States have LS, while 1 in 6 patients with early-onset colorectal cancers (below the age of 50) is associated with LS (Sinicrope, 2022).

With the impressive clinical outcomes and recent approval of immune checkpoint inhibitors (ICIs) in the treatment of metastatic MSI-H cancers irrespective of organ of origin, we are seeing an increasing convergence in their treatment strategies with a “systemic treatment first” strategy being increasingly adopted. Given the similar genotype and mutator phenotype, MSI-H tumors often display certain similarities in their clinical trajectory and tumor biology, though key differences exist. We summarize in this review the updated clinical, genomic, and tumor immunobiology of MSI-high cancers, with specific attention to the site of metastatic or recurrent disease, treatment options, and clinical outcomes.

## Definitions and detection of microsatellite instability

Microsatellites are 10–60 base pair (BP) length tandem repeats of between 1–6 nucleotides and are widely distributed, non-randomly, but predominantly in the non-translated and non-transcribable regions across the human genome. As mentioned previously, defective mismatch repair genes result in the formation of slippages in DNA replication over areas of microsatellites, leading to either insertion or deletion (Indels) of the nucleotide repeats and, hence, instability, or polymorphisms, of microsatellite regions. Microsatellite instability in functional genes (introns, non-coding exons, and coding regions), especially those in the coding regions, are associated with multiple conditions, including hereditary disorders and cancers, and have been thought to contribute to phenotypic plasticity through gene regulation and the transcription/protein function (Bagshaw, 2017).

MSI-H-related Indels at the non-coding regions may have a limited impact but those at splicing-required segments may affect gene expression, and Indels at the coding regions can lead to frameshift mutations, leading to driver mutations that are implicated in carcinogenesis. It also produces an array of novel neoantigens (with multiple immunogenic neoepitopes) and the previous encoded protein truncated, resulting in a loss of function of key tumor suppressor genes (TSGs). MSI-high tumors have characteristically a high tumor mutation burden (TMB) and a somatic mutational pattern that is distinct across tumor types. Inferring from the mutator hypothesis, the MSI phenotype may lead to acquired mutations in other key DNA repair pathway genes, such as the double-stranded break (DSB) repair and base excision repair pathway, leading to further accumulation of somatic mutations, accounting for high TMB and further events of tumorigenesis.

The current gold standard for MSI detection is a fluorescent multiplex polymerase chain reaction and capillary electrophoresis (PCR-CE) of five specific loci (BAT25, BAT26, D2S123, D5S346, and D17S250), where more than 30% variations in the lengths between that in tumor and normal is defined as microsatellite instability (NCI Panel) (Boland et al., 1998). MSI-H is also detected by other techniques, such as next generation sequencing (NGS)–based algorithms with high sensitivity and specificity (Jass, 2007; Cheng et al., 2017; Bonneville et al., 2020). NGS testing can simultaneously detect specific somatic mutations and TMB. Multiple platforms have been validated to be able to detect MSI-H in somatic tumors, for example, MSKCC-IMPACT showed 92% uniformity results in their MSI testing, and the Foundation Medicine Inc. (FMI) platform MSI testing has also been approved recently as well. Other examples of such algorithms include mSINGS, MSISensor, and MANTIS (Li et al., 2020). A recent study demonstrated that MANTIS achieves high sensitivity (97%) and specificity (99%) across six cancer types and provides stable performance with varying numbers of microsatellite loci. We foresee NGS being incorporated routinely in clinical practice across multiple tumor subtypes in the future and, hence, a need to harmonize the various platforms of MSI testing (Table 1).

**TABLE 1:** Comparing clinical, histologic and genomic features of MSI-H colorectal, gastric, and endometrial cancers.

Comparing MSI-H colorectal, gastric, and endometrial cancers				
		Colorectal	Gastric	Endometrial
Clinical Features	Age	Associated with younger patients	Associated with older patients (≥ 65yo)	
	Stage at Presentation	Presents at an early stage	Presents at an early stage (less frequent lymph node involvement and less invasiveness)	Presents at a late stage
	Location	Proximal or right colon	Middle/lower gastric body	
	Invasiveness	Deep infiltrative	Less invasive	Deep myometrial invasion
	Grade	Poorly differentiated	Poorly differentiated	Higher grade
	Sites of recurrence	Local recurrence and peritoneal metastases most common	No difference	Peritoneal recurrence most common
Histological Features	Histological subtype	Serrated adenocarcinoma, medullary or mucinous	Mucinous or intestinal	Endometrioid
	Immune cell infiltration	Rich immune cell infiltration intra- and peritumourally	Prominent lymphocyte infiltration	Lymphocyte infiltration
Genomic Features	Underlying genomic mutations	Transcriptional silencing of hMLH1 (sporadic MSI-H); germline MMR gene mutations (Lynch Syndrome) High mutational burden, high frameshift mutation events, relatively lower SNV events compared to MSS; immune-related gene signatures, oncogenic and DNA repair pathways can be affected across MSI-H cancers		
		Frameshifting DNA slippage events are the primary inactivating mechanism of hMSH3 and hMSH6		Frameshifting DNA slippage events are the primary inactivating mechanism of hMSH3 and hMSH6
		TGFB related genes such as TGFBR2 and ACVR2A, apoptotic regulator BAX are particularly susceptible to SNV and microsatellite mutations; somatic BRAF association with MLH1 promoter hypermethylation	BAX susceptible to SNV and microsatellite mutations; ACVR2A and ORC4 susceptible	Low ORC4 and ACVR2A mutations, higher PTEN mutations
Germline association	Most associated genes	MLH1 and MSH2	Unclear	Unclear

As a broad definition based on the NCI panel, we refer to patients with two or more microsatellite loci instability as MSI-high (MSI-H). Tumors with one out of five microsatellite instability loci are classified as MSI-Low (MSI-L), and a lack of microsatellite instability in any loci classified as microsatellite stable (MSS), though in most settings the MSI-L and MSS subtypes are grouped as MSS. One group also reported that MSI-L and elevated microsatellite alterations at selected tetranucleotide repeats (EMAST) are prognostically worse than that of MSI-H and are distinct also from MSS subgroups (Garcia et al., 2012).

MSI testing requires paired tumor and normal DNA, which is not typically available for core tumor biopsy. For the latter, immunohistochemical staining of the four MMR proteins to detect their presence (pMMR) or deficiency (dMMR) in tumor is used, with a high concordance rate with sequencing-based tests for MSI-H. Liquid biopsy for MSI-H testing using cell-free plasma-circulating DNA (cfDNA) is currently in development and show potential for clinical use in the future, especially in early cancer detection and surveillance post definitive treatment (Tieng et al., 2021).

## Clinical presentation and histologic characteristics

In view of the rarity of other MSI-H cancers, we will focus on MSI-H colorectal, gastric, and endometrial cancers. Sporadic MSI-H colorectal cancers (CRCs) are more common compared to germline MSI-H cancers and arise from the hypermethylation of the promoter region of MLH1. In general, MSI-H CRCs present in younger patients, with early-stage disease, right sided or proximal colon tumors. The tumors are generally poorly differentiated (higher grade), serrated adenocarcinoma, deep infiltrative tumor with medullary or mucinous subtype and rich immune cell infiltrates intratumorally and peritumorally (Kim et al., 1994; Jass, 2007). In the metastatic setting, MSI-H CRC often presents with synchronous metastases involving the lymph nodes and intra-abdominal disease rather than distant metastases. One case series (*n* = 129) also showed distinct patterns between sporadic and inherited cases of MSI-H CRC, suggesting heterogeneity among these tumors based on the underlying etiology of MMR deficiency (Cohen et al., 2017). LS patients, or patients with germline mutations, were associated with more frequent liver involvement, metastatic resection, and better disease-free survival after metastasectomy (HR = 0.28, *p* = 0.01) compared to sporadic cases (Cohen et al., 2017).

MSI GCs have been associated with an older age (more or equal to 65 years), female gender, tumoral location in the middle/lower gastric body, less frequent lymph node involvement, and less invasiveness (Polom et al., 2018; Zubarayev et al., 2019). Similarly, typical histological features include the association with mucinous or intestinal subtype GC with prominent lymphocyte infiltration, similar to MSI-H CRC. One recent study suggests the use of deep learning in recognition of MSI-H gastrointestinal tumors *via* H&E staining, highlighting their strong similarities (Kather et al., 2019). In endometrial cancers, the MSI-H phenotype is more common in the endometrioid subtype and is associated with a higher tumor grade, deep myometrial invasion, and higher clinical stage (Kanopiene et al., 2014), but some other studies did not show significant correlation.

The MSI-H status has been increasingly investigated and found to be a positive prognostic marker in various cancer subtypes (Kanopiene et al., 2014; Pietrantonio et al., 2019). The MSI-H status has been well studied in colorectal cancers and was found to be a positive prognostic and predictive factor for immune checkpoint inhibitors. In terms of a predictive marker for chemotherapy, studies have shown that stage II colorectal cancers with the MSI-high phenotype do not benefit from 5-fluorouracil (5FU) chemotherapy (Sargent et al., 2010), although the addition of oxaliplatin to 5FU in the adjuvant setting was found to prolong survival in comparison to surgery alone (Green et al., 2020). A meta-analysis of patients with gastric cancer found that patients with MSI-H tumors had worse pathological treatment response to neoadjuvant chemotherapy but better overall survival than the MSS tumor cohort (Vos et al., 2021). A new ESGO ESTRO ESP 2021 classification for endometrial cancer has now included mutational analysis of POLE, MMR, and p53 immunohistochemistry testing in line with TCGA molecular-based classification for endometrial cancers. POLE-mutated tumors (hypermutated tumors) were associated with the best prognosis, p53-mutated tumors with worst prognosis, and dMMR/non-specific molecular profile with intermediate prognosis. Overall, the prognostic advantage of the MSI-H status in early-stage endometrial cancers is not clear (Concin et al., 2021; Vos et al., 2021).

## Familial syndromes

Patients with MSI-H/dMMR cancers are recommended to undergo genetic counseling and germline MMR testing. Again, except for MLH1 loss with BRAF V600E mutation, which predicts for sporadic MSI-H CRC, MMR protein loss and MSI-H status is a strong indication for association with Lynch Syndrome. Patients with LS have monoallelic MLH1-, MSH2-, MSH6-, and PMS2- inactivating mutations and a secondary acquired somatic mutation; gremline biallielic MMR gene mutations can lead to very early onset LS cancers, and they may often present multiple early-stage cancers (colorectal, endometrial, gastric, biliary tract, pancreatic, small bowel, and gastric cancers and cancer at other sites such as ovarian, prostate, bladder, thyroid, breast, and CNS cancers and leukemia) through detected symptoms or regular screening (physical examination, laboratory investigations, imaging, and/or specialized tests such as endoscopes), with recurrence rates generally low after completed resection. A second primary may develop following primary colon cancer, either with another colonic primary or LS tumor of other sites.

The Amsterdam criteria were devised to identify family history suspicious of HNPCC. The criteria are defined by at least three individuals with CRC, two successive generations with one individual being a first degree relative of the other two and at least one diagnosis before age 50, while the Amsterdam criteria II includes extra-colonic tumors such as endometrial cancer. The Revised Bethesda II criteria or Amsterdam criteria II is not sufficient for detection/diagnosis of LS, and a formal MMR/MSI testing is required. For germline pathological MLH1 or MSH2 carriers, surveillance colonoscopies should be initiated at the age of 25 years. For pathological MSH6 or PMS2 carriers, surveillance colonoscopies should be initiated at the age of 35 years (Seppälä et al., 2021). Chromoendoscopy of the proximal colon has been found to be inferior to white light endoscopy in the initial detection of neoplasia in LS screening. There was no consensus on the screening of other LS cancers. For female patients with LS, ESGO/ESTRO/ESP guidelines also recommend that surveillance should generally start by age 35 but with personalized programs, *via* annual transvaginal ultrasound (TVUS) and annual or biennial biopsy until hysterectomy is performed. Hysterectomy and bilateral salpingo-oophorectomy may be performed at the completion of childbearing and preferably before the age of 40, with benefits and risks discussed about prophylactic surgery.

The largest LS databases published to date followed up 1942 mutation carriers without previous cancer for 13,782 observation years. In total, 314 patients developed cancer, mostly colorectal (*n* = 151), endometrial (*n* = 72), and ovarian (*n* = 19). Cancers were detected from age 25 onwards in MLH1 and MSH2 mutation carriers and from about age 40 in MSH6 and PMS2 carriers. Among first cancer detected in each patient, the colorectal cancer cumulative incidences at 70 years by gene were 46%, 35%, 20%, and 10% for MLH1, MSH2, MSH6, and PMS2 mutation carriers, respectively. The equivalent cumulative incidences for endometrial cancer were 34%, 51%, 49%, and 24% and 11%, 15%, 0%, and 0% for ovarian cancer (Møller et al., 2017).

## Heterogeneous genomic characteristics

The sporadic MSI-H phenotype most commonly occurs from hMLH1 promoter region (CpG) hypermethylation or transcriptional silencing. Although most MSI-H CRC, gastric, and endometrial cancer genomes harbor transcriptional silencing of hMLH1, frameshifting DNA slippage events are the primary inactivating mechanism for hMSH3 and hMSH6 in MSI-H CRC and endometrial cancer genomes. Other MMR genes, such as hMSH2, hPMS1, and hPMS2, harbor nonsilent (missense or nonsense) single nucleotide variations (SNVs), mostly in the hypermutated samples (Kim et al., 2013). Interestingly, nonsilent SNVs and DNA slippage events are mutually exclusive for both hMSH3 and hMSH6 in MSI-H genomes, suggesting that these two may be alternative mechanisms for inactivation (Ciriello et al., 2012).

Studies have demonstrated that MSI subtypes are more nuanced than the discrete classification commonly used and that there exists significant heterogeneity within the MSI-H phenotype. For example, significant molecular heterogeneity has been observed in multiple subtypes of MSI-H phenotypes: biallelic vs. monoallelic germline MMR deficiency, hereditary Lynch Syndrome vs. sporadic MSI-H phenotype, and variability across the aforementioned MMR genes. An example of this heterogeneity was the higher level of TMB observed in MSI-H CRC with MSH2 or MSH6 mutations compared to MSI-H CRC with MLH1 or PMS2 mutations (Battaglin et al., 2018). Furthermore, novel mutations such as NTRK fusions have been found to be associated with MSI-H CRC, pointing to potentially new and unique subgroups under MSI-H (Wang et al., 2022). It has been demonstrated that even in Lynch Syndrome significant heterogeneity exists to the extent that it can be divided into 2 groups, G1 and G2, based on their genomic characteristics. G1 demonstrates greater mutational frequency and MSI compared to G2. At the same time, G1 also demonstrated notable differences with MSI-H tumors, such as not commonly presenting with BRAF mutations. Abnormal KRAS signaling was also seen in G1, which is also often seen in MSS tumors (Binder et al., 2017).

The localization of MSI events is suggested to be influenced by chromatin configuration. This was demonstrated by Binder et al., which highlighted the lack of MSI events at stable nucleosome positions and, contrastingly, the abundance of MSI events at the euchromatic regions. MSI event density is also demonstrated to be reduced in regions with a larger number of SNVs or point mutations (Cortes-Ciriano et al., 2017). Also, MSI frameshift mutations affect not only coding regions but also non-coding regions. This results in disruptions to normal miRNA production and gene regulation. It has been demonstrated that MSI and MSS tumors express miRNA differently, although this discrepancy has not been established to have a prognostic value (Randrian et al., 2021).

Consensus molecular subgroup (CMS) classification suggests MSI-H colorectal cancers to be part of CMS subtype 1, together with BRAF V600E and the CpG island methylator phenotype (CIMP), and a high immune cell infiltration in the stroma. Similarly, data from TCGA database, in combination with other studies, have allowed subclassification of gastric, endometrial, biliary tract, and pancreatic cancers into distinct molecular classifications—the MSI-high phenotype being a commonality. MSI-hypermutated type of endometrial cancer is characterized by a high mutation rate (18 × 10^6^ mutations/Mb) and over-expressed immune-related gene signatures and biomarkers (Vanderwalde et al., 2018). Similar characteristics were seen in MSI-H gastric and other gastrointestinal cancers.

Overall, MSI-H cancers share more similarities in their genomic mutational landscape with their counterparts in other solid organ tumors, as compared with their MSS counterparts in the same organ. They share common microsatellite loci mutations and a high mutational burden, characterized by frameshift mutations from indels in both the coding and non-coding regions and generally lower SNV mutations (Gatalica et al., 2016). Le et al. reported a mean of 1782 somatic mutations per tumor in patients with dMMR cancer (N = 9), as compared with 73 mutations per tumor in patients with pMMR cancer (N = 6) (*p* = 0.007).

A study summarized genomic data from MSI-H tumors from multiple cancer types and found that several DNA repair pathways other than MMR, including ataxia telangiectasia and Rad3-related (ATR), base excision repair (BER), homologous recombination (HR), and non-homologous end joining (NHEJ), are altered by SNV and microsatellite mutations. Along with the diverse molecular functions enriched for MSI events in these tumor types, some genes (TGFBR2, ACVR2A, and BAX) are particularly susceptible in MSI-H CRCs (Markowitz et al., 1995; Rampino et al., 1997; Jung et al., 2004), and BAX is also susceptible in MSI-H gastric cancers.

For instance, TGF-β receptor 2 (TGFBR2) was the first MSI gene to be identified, and several frameshift mutations leading to loss of function in this gene are seen in 80% of MSI-H CRCs, while less commonly so in endometrial cancers (Randrian et al., 2021). This suggests that the loss of function of TGFBR2 confers a survival advantage for cancer cells during tumorigenesis for MSI-H CRCs, but this is less evident in MSI-H endometrial cancers. TGFBR2 frameshift mutations also result in the generation of possibly immunogenic neoantigens, TGFBR2 signaling impairment may also directly promote inflammation in the tumor microenvironment of CRC. Evidence suggests that MSI-H tumors have a higher propensity of generating neoantigens, with up to 30% of mutations in MSI-H tumors predicted to result in neoantigens (Randrian et al., 2021). An understanding of the mutational landscape, shaped by genomic instability as a hallmark of these cancers, has also led to better understanding of the driver mutations, tumor biology, and tumor-immune microenvironment, which will be discussed further.

However, the functional relationship between MSI and tumourigenesis and the similarity of molecular mechanisms that establish the MSI phenotype across cancer types remain to be validated. The genes frequently targeted by MSI in CRC genomes, as mentioned previously, usually do not harbor DNA slippage events in endometrial cancer genomes (Gurin et al., 1999), and it is largely unknown whether MSI-H endometrial cancer mutations have similar molecular origins or functional consequences as CRC mutations. Hause et al. analyzed 5,930 cancer exomes from 18 cancer types from TCGA database and combined more than 200,000 microsatellite loci (using a novel MSI detection tool, MOSAIC) to construct a genomic classifier for MSI (Hause et al., 2016). An example of differentially unstable microsatellites included important genes in oncogenesis such as NIPBL, TCF4, and PTEN. An example is also in the microsatellites in ACVR2A (related to the TGF-β pathway) and ORC4: the former was unstable in 90% of colon, 67% of rectal, and 87% of gastric MSI-H tumors but only 28% of endometrial MSI-H tumors; the latter was unstable in 97% of colon, 67% of endometrial, and 100% of rectal and stomach MSI-H tumors investigated. There was further suggestion that these specific instability signatures of cancer-associated genes reflect selective pressures and can potentially identify novel cancer drivers. Specific gene clusters (immune-related, cell stress response, DNA damage, chromosome-related, and transmembrane/TGF-β) (Cortes-Ciriano et al., 2017) are noted to have recurrent MSI, many with tumor type-specificity. Interestingly, despite differences in other mutational signatures, genes functionally involved in immune regulation are significantly enriched in both MSI-H CRC and endometrial cancers (Kim et al., 2013).

## Immunobiology of MSI-H cancers

Frameshift peptide (FSP) repertoire of MSI-H cancers and their interaction with host immune cells are key elements in dictating the immune equilibrium and the initial “elimination phase” that precede the disease. For example, FSP-specific T-cell responses are detectable in peripheral blood of asymptomatic Lynch Syndrome mutation carriers. Also, LS patients are found to have thousands of microscopically small premalignant lesions comprising single MMR-deficient crypts (Kloor and von Knebel Doeberitz, 2016). However, this tumor elimination or immune surveillance is not always efficient due to eventual immune escape that resulted from selection pressure from host.

In view of the high frameshift peptide (FSP)-based antigenic load of MSI-H cancers driven by genomic instability, the tumor-immune microenvironment is typically heavily infiltrated by lymphocytes and innate immune cells, though the T-cell exhaustion phenotype and upregulation of resistance and co-inhibitory pathways are commonplace. Tumor immunogenicity from frameshift mutations occur through the novel immunogenic FSPs that are presented by major histocompatibility complex (MHC) class-I molecules on tumor cells and by MHC class-I and II on antigen-presenting cells (APCs), being recognized as non-self. There is strong T-cell priming and activation when presented by APCs in nearby lymph nodes or tertiary lymphoid structures and, hence, increased trafficking of effector T cells and other immune cells into the tumor microenvironment. These FSPs, despite showing temporal changes in their repertoire, often display commonalities across MSI-I subtypes (Kloor and von Knebel Doeberitz, 2016) and again make them good targets for potential therapeutics including vaccine strategies. Separately, the DNA fragments from the defective DNA mismatch repair pathway can also elicit an innate immune response through the DNA sensor, cGAS-STING pathway. The single- or double-stranded DNA fragment may be recognized as non-self, damage-associated molecular pattern (DAMP), leading to a type I interferon release and transcription of multiple downstream interferon-related genes/pathways. This again results in increased immune activation and both innate and adaptive immune response in these tumors.

Heterogeneity in both tumor biology and the immune microenvironment has resulted in MSI-H cancers having generally strong, but at times variable, response to ICIs. This will be mentioned in greater detail in the subsequent sections. One study suggests that non-responders to ICIs have been found to have a more strongly immunosuppressive TME, with upregulation of pathways such as Wnt/β-catenin, KRAS, and TGF-β. In contrast, responders demonstrated upregulation of IFN-γ, pointing to a more immunoreactive profile (Chida et al., 2022). Patterns of resistance seem to be predominated in pathways of antigenic presentation such as in the HLA and B2M gene mutations, while mutations in the Wnt/β-catenin pathways result in reduced T-cell infiltration (Grasso et al., 2018). A separate study also implicated JAK1 mutations and the loss of the associated interferon response as a possible mechanism of immune evasion (Albacker et al., 2017).

As mentioned previously, it is known that mutations in B2M would impact tumor neoantigen presentation and immune activation (Argyropoulos et al., 2017) B2M-mutated CRCs have been shown to be associated with higher levels of PD-1 positive T cells, pointing toward potential PD-1 inhibitor resistance (as has already been observed in melanoma) (Janikovits et al., 2018). Mutations in another protein, HSP110, have also been associated with improved prognosis (Oh et al., 2017). Expression of certain immune checkpoint ligands on T cells, specifically CD274, LAG3, and IDO1, have also been found to be associated with lower rates of recurrence following surgery (Lee et al., 2018). On the other hand, mutations in SMAD4, a tumor suppressor gene, were found to be associated with a poorer prognosis in MSI-H CRCs but was not associated with survival in MSS CRCs (Isaksson-Mettavainio et al., 2012).

## Treatment strategies in stage II and III MSI-H cancers

In terms of early-stage MSI-H cancers, the most reported cancers are colorectal cancers, in part due to routine MSI/MMR testing. Surgical resection alone or combined with adjuvant chemotherapy remains the cornerstone of treatment for non-metastatic MSI-H CRC (André et al., 2004; Taieb et al., 2019). While the standard segmental colorectal resection is the most common operation performed for MSI-H colorectal cancers, extended resection—either subtotal colectomy with ileosigmoidal anastomosis or total colectomy with ileorectal anastomosis—may be considered based on the specific MMR gene variant and the associated risk of metachronous colorectal cancer. Extended colorectal resection should be considered for dMMR patients with a metachronous colorectal cancer and may be considered for patients with germline hMLH1 or hMSH2 deficiency with primary CRCs to reduce the risk of developing subsequent colorectal cancer (Seppälä et al., 2021). The extent of colorectal resection should be individualized based on patient’s age, gender, specific dMMR genetic variants, and anticipated postoperative bowel function.

It is clear from multiple retrospective cohort studies that patients with MSI-H colorectal cancers have better stage-adjusted survival than their microsatellite-stable (MSS) counterparts (Halling et al., 1999; Gafà et al., 2000; Samowitz et al., 2001; Lanza et al., 2006; Sinicrope et al., 2006). Superior oncological outcomes of MSI-H tumors are more apparent in earlier stage tumors (Roth et al., 2012). In particular, the survival advantage of MSI-H tumors appears to be greater among stage II compared to stage III CRC patients. A recent retrospective review of epidemiology data from the American College of Surgeons Commission on Cancer included 16,788 stage II CRC patients of which 1709 were microsatellite unstable (Cavallaro et al., 2021), from 2010 to 2016. MSI-H cancers with high-risk features were found to have significantly better overall survival than MSS cancers with high-risk features and had survival similar to MSS cancers with low-risk features. The incidence of MSI-H colorectal cancers is higher among stage II compared to stage III CRC (20% vs. 12%) and are relatively uncommon among metastatic tumors (4%) (Roth et al., 2010). In addition, MSI-H stage II CRC are potentially more likely to be cured by surgical resection alone, given the favorable prognostic value conferred by the MSI-H status and a lack of any benefit from adjuvant 5FU-based chemotherapy after surgery.

The majority of early-stage MSI-H gastric cancers are located in the distal third of the stomach, with intestinal pathology. MSI-H GCs have been associated with a high rate of N0 stage, lower number of lymph node metastases, and a less extensive spread to lymph node stations, compared to their microsatellite-stable counterparts. However, studies have been limited by small numbers. Currently, D2 lymphadenectomy for GC is the standard of care based on superior local control and better overall survival. For stage III CRC and gastric cancers, adjuvant chemotherapy with oxaliplatin and fluoropyrimidines is recommended irrespective of the MSI status, in view of higher stage of cancer and benefit of chemotherapy based on overall cohorts (Tougeron et al., 2016). Surgical consideration for endometrial carcinoma is independent of MSI-H status currently, with oncological total hysterectomy and bilateral salpingo-oophrectomy. Adjuvant chemotherapy is recommended for high-grade endometrial cancers, and until recently, the molecular status has not directly affected the decision for adjuvant chemotherapy and/or radiotherapy (Green et al., 2020). With the recent ESGO recommendations, adjuvant therapy for endometrial cancers may also be influenced by not just the histological grade but the molecular profiling and hence genomic risk of recurrence for the patients.

With continual improvements in surgical techniques, the boundaries of surgical resectability are constantly expanding. One meta-analysis showed that amongst patients with MSI-H tumors, neoadjuvant/adjuvant chemotherapy did not have any effect on overall survival (Møller et al., 2017). Neoadjuvant studies in gastric and colorectal cancers showed poor pathological responses in MSI-H subtypes, suggesting the potential of combining immunotherapy with chemotherapy in the neoadjuvant setting in the context of clinical trials, which will be discussed in subsequent sections (Seymour et al., 2019). We foresee MSI and molecular testing being carried out routinely for locally advanced gastrointestinal cancers in the future, especially in gastric and colorectal cancers, which will better inform neoadjuvant and adjuvant therapy.

Currently, prophylactic colorectal resection for germline dMMR patients in the absence of colorectal cancer is not recommended. Prophylactic hysterectomy may be discussed pre-operatively for female LS patients with early-stage colorectal cancers. Similarly, prophylactic salpingo-oophrectomy can be discussed in LS patients who have completed childbearing. Generally, recurrence rates are low in early-stage MSI-H tumors, presumably in part due to host immunosurveillance and the underlying tumor biology. Hence surveillance is individualised, but with specific attention to LS patients who may develop another second primary LS cancer.

## Metastatic and recurrence distribution

We summarize three studies that looked at recurrence patterns in MSI-H and MSS CRC and gastric cancer, respectively. There was a lack of clinical data on endometrial cancers and other tumors, presumably due to the low case numbers and/or lack of MSI/MMR testing in early-stage setting (Table 2) (Sinicrope et al., 2011; Kim et al., 2016; An et al., 2020).

**TABLE 2:** Sites of recurrence for early-stage MSI-H colorectal and gastric cancers.

Colorectal cancer										
Kim et al. (2016)										
			*N*		5-Years DFS		HR (95% CI)		p value	
MSI status		MSI-H	251		91.10%		0.0619 (0.508-0355)		<0.001	
		MSI-LIMSS	2679		78.20%		1			
Site of recurrence		All patients	MSI-H		%		MSS		%	
Local recurrence		51	6		11.76%		45		88.24%	
Peritoneum		54	8		14.81%		46		85.19%	
Ovary		17	0		0.00%		17		100.00%	
Hematogenous^a^		369	6		1.63%		363		9837%	
Gastric cancer											
An et al. (2020)											
				*n*		5-year survival		HR		p value	
MSI status		MSI-H/dMMR		64		4.69%		1.155 (0.005-1306)		0.29	
		MSS/pMMR		726		4.13%					
Site of recurrence		All patients		MSI-H		%		MSS		%	
Local recurrence		132		11		833%		121		91.67%	
Peritoneum		390		33		8.46%		357		9154%	
Ovary		43		2		4.65%		41		95.35%	
Hematogenous		154		11		7.14%		143		92.86%	
Colorectal cancer											
Sinicropc et al. (2011)											
	Proficient MMR (*n* = 1797)				Deficient MMR (*n* = 344)						
Variable	No. (%)				No. (%)				p value		
Recurrence status									<0.001		
No recurrence	1222 (81.8)				271 (18.2)						
Recurrence	575 (88.7)				73 (11.3)						
Site of recurrence									0.171		
Intra-abdominal	122 (84.7)				22 (15.3)						
Local only	41 (91.1)				4 (8.9)						
Distant	387 (90.2)				42 (9.8)						

^a^ Combined values for the lung, extra-abdominal Iymph nodes, bone, muscle, brain, and liver.

The pattern of recurrence of MSI-H colorectal cancers has been reported to be different from that of microsatellite-stable colorectal cancers. From one study comparing stage I-III MSI-H and MSS colorectal cancers that have recurred, MSI-H colorectal cancers more frequently exhibited local recurrence (30.0% vs. 12.0%, *p* = 0.032) and peritoneal metastases (40.0% vs. 12.3%, *p* = 0.003), compared to patients with recurrent MSI-L/MSS CRCs. In addition, MSI-H CRCs more frequently recurred as isolated peritoneal (25.0% vs. 3.7%, *p* = 0.001) or intra-abdominal lymph node metastases (15.0% vs. 3.7%, *p* = 0.048). In contrast, lung and liver metastases (hematogenous spread) were more frequent among patients with MSI-L/MSS CRCs. Another case series from a korean group on MSS/pMMR (*n* = 726) vs. MSI-H/dMMR (*n* = 64) resected gastric cancer did not show a significant difference in sites of recurrence (*p* = 0.816) though majority of patients had peritoneal recurrence in both groups (Sinicrope et al., 2011; Kim et al., 2016; An et al., 2020).

A study showed that the concordance of MSI and MMR status in primary CRC and corresponding metastatic cancer is potentially organ-specific. High concordance is found in liver, lung, and distant lymph node metastases, whereas discrepancy is more likely to occur in peritoneal or ovarian metastasis (Cheng et al., 2017; He et al., 2019). This may again suggest that there are differences in the disease biology for peritoneal metastases, compared against metastases from hematogenous spread in recurrent metastatic MSI-H cancers. Immune-escape mechanisms and/or further tumor evolution may be at play and we seek further studies for clarification. The recurrence pattern for endometrial cancers has not been well described but intra-abdominal recurrence is also a key recurrence pattern regardless of MSI status. More commonly described for MSI-H/dMMR endometrial cancers are detection of second primary, where colorectal primary is the most common site (Post et al., 2021).

One MSI-H mCRC (*n* = 502) study showed that inferior outcomes were observed in patients with peritoneal metastases and ascites (aHR 2.90, 95% CI 1.70 to 4.94; aHR 3.33, 95% CI 1.88–5.91) compared with patients without peritoneal involvement. The mGC (*n* = 59) cohort showed inferior PFS and OS in patients with peritoneal metastases and ascites (aHR 3.83, 95% CI 1.68 to 8.72; HRaHR 3.44, 95% CI 1.39 to 8.53, respectively) when compared both with patients with peritoneal metastases (no ascites) and patients without peritoneal involvement (Fucà et al., 2022).

## Treatment considerations in stage IV MSI-H tumors

Survival outcomes of MSI-H cancers prior to immune checkpoint inhibitors were poor, before the era of immunotherapeutics, owing to poor chemo-responsiveness of these cancers. This has brought immunotherapy, especially immune checkpoint inhibitors (ICIs) to the frontline or second line treatment of metastatic MSI-H/dMMR cancers. ICIs such as anti-PD-1 and anti-CTLA-4 therapies have shown dramatic responses in MSI-H cancers and prolongation of progression free survival. Interestingly, the use of ICIs in the neoadjuvant space is now increasingly explored with translational readouts that will shed light on biomarkers of response/resistance. Given the opportunity for tumor specimen collection and pre- and posttreatment comparison, neoadjuvant ICI studies in MSI-H cancers are key in both understanding the clinical response as well as the tumor-specific biomarkers of response and pattern of treatment responses in both the tumor and the tumor microenvironment.

A key phase IIB trial, Keynote 164, first confirmed the efficacy of pembrolizumab in the second line setting in multiple MSI-H solid organ tumors, paving the way for a tumor-agnostic, genomic profiling–based therapeutic paradigm (Le et al., 2020). Nivolumab has also been approved for use in MSI-H colorectal cancers, based on an overall response rate (ORR) of 40% in a phase 2 Checkmate study. Keynote 158 (O’Malley et al., 2022), which includes 233 patients with one of 27 different advanced MSI-H/dMMR non-CRC solid organ cancers, received pembrolizumab for up to 2 years, with 33.4% showing objective response, but ORR varied substantially when stratified by tumor histology–pancreatic cancer with 18.2% ORR, CNS tumors without any response and endometrial cancers with 57.1% ORR. Generally, patients with MSI-H endometrial, gastric, ovarian and small intestine cancers performed well and median OS was not reached at the time of reporting, while patients with cholangiocarcinoma also reached 24.3 month mOS. Keynote 177, a phase 3 trial which compares pembrolizumab with standard of care chemotherapy in first-line setting for stage IV MSI-H/dMMR colorectal cancers, reported a high response rate (43.8% vs. 33.1%), and a significantly improved median progression free survival of 16.5 months *versus* 8.2 months, with HR of 0.59 (95% CI 0.45–0.79) (Diaz et al., 2017). Again, we consider pembrolizumab to be suitable for frontline setting in advanced MSI-H/dMMR CRC but look forward to future combinatory options that may further improve response and duration of response. The treatment paradigm of MSI-H gastric cancers and endometrial cancers are less well defined, but with second line option of pembrolizumab given the data from Keynote 164. Again, further combinatory studies are awaited to improve response and survival.

Neoadjuvant or systemic treatment-first approach is increasingly considered in the context of stage IV and locally advanced MSI-H/dMMR cancers. This is considering the impressive clinical, radiological and pathological response to immune checkpoint blockade. A recent phase II trial undertaken by MSKCC showed a highly impressive response rate of 100% in patients with dMMR/MSI-H locally advanced rectal adenocarcinoma, albeit with a relatively short duration of follow up (Cercek et al., 2022).All patients had gone into complete clinical response (*n* = 12) and have not undergone either neoadjuvant chemoradiation or surgery. The Niche study of neoadjuvant ipilimumab and nivolumab in locally advanced colon cancers also demonstrated the impressive major pathological response (19 out of 20 patients) (Vos et al., 2021). This highlights a very interesting hypothesis that to introduce ICI early in the evolution of MSI-H cancers may be important, and may allow immune mediated tumor elimination before subsequent resistance pathways develop overtime that may have led to the advanced disease. The tumor microenvironment in the primary organ *versus* the sites of metastases may also differ significantly, as data suggests metastatic sites have differing response to ICIs. A more tailored approach may be required in the future, especially for patients with MSI-H cancers, in the neoadjuvant setting, however in the context of clinical trial currently due to the paucity of data on survival outcomes in the abovementioned trials.

In the context of metastasectomy and peritonectomy, a more holistic approach should be taken to reduce morbidity and risks to patients with MSI-H cancers. One concern, however, remains to be the non-responders, pseudo-progressors and hyper-progressors in patients who have been treated with immune checkpoint inhibitors in the frontline setting. In the context of MSI-H CRC, patients who have borderline resectable oligometastatic disease will need to be counseled with regards to the risk and benefit of neoadjuvant immunotherapy, as there is a risk of progression, as evidenced by the early progressors in the Keynote 177 study (André et al., 2020). However, for the responders, upfront immunotherapy affords the chance of good partial response or even complete response, negating the requirement for invasive surgery. The role of surgical debulking and metastasectomy in MSI-H CRCs may be increasingly challenged in the era of effective systemic therapy such as immune checkpoint inhibitors and other novel therapeutics.

One concern, however, remains to be the non-responders, pseudo-progressors and hyper-progressors in patients who have been treated with immune checkpoint inhibitors in the frontline setting. In the context of MSI-H CRC, patients who have borderline resectable or resectable oligometastatic disease will need to be counseled with regards to the risk and benefit of neoadjuvant immune checkpoint inhibitors, as there remains a risk of progression to unresectability, as evidenced by some early progressors in the pembrolizumab arm in the Keynote 177 study. However, for the responders, upfront immunotherapy affords the chance of good partial response or even complete response, negating the requirement for invasive surgery, though durability of response on immune checkpoint inhibitors remain to be seen.

We noted from aforementioned studies that peritoneal disease in recurrent/metastatic MSI-H cancers are common and may exhibit a slightly different disease biology as seen in the discordance of dMMR/MSI-H status from the primary tumor. This may suggest a role of peritoneal biopsy for patients with peritoneal recurrence in MSI-H cancers to understand the molecular status and tumor biology of advanced MSI-H (and MSS) CRC for more holistic treatment strategies. The role of optimal surgical debulking and peritonectomy for peritoneal-limited gastrointestinal cancers and gynecological cancers has been debated, and will remain contested for MSI-H tumors. Also, one study showed that ascites with peritoneal disease is also a poor prognostic marker, as opposed to peritoneal disease only or metastases to other sites—suggesting that the selection of surgical candidates should be judicious.

Despite the emerging clinical trials in immune checkpoint inhibitors against MSI-H cancers, the overall response rates across various tumor types typically do not exceed 50% in stage IV setting, as a stark contrast to the high response to locally advanced, non-metastatic MSI-H colorectal cancers which have reported clinical and/or pathological response rates of >90%. This raises the question again of acquired evasion and resistance pathways that may need to be overcome (or perhaps circumvented by initiating immunotherapy at an early stage), to improve treatment and patient outcomes.

Our centre is also focusing on a unique group of cancers with carcinomatosis peritonei and looking deeply into the molecular and oncologic drivers of this group of cancers. We believe that emerging data on MSI-H cancers will continue to shed light on the appropriate management strategies for this unique class of tumors. Again, though immune checkpoint inhibitors remain a point of convergence for advanced MSI-H cancers, differing somatic mutations, tumor-immune microenvironment, tumor/organ-specific intrinsic factors and sites of metastases will continue to require tailored approach.

## Novel therapies and biomarkers of response/resistance in MSI-H cancers

MSI-H CRCs are often immune cell rich as mentioned, but the TILs may exhibit an activated and/or exhausted phenotype (higher expression of TIGIT, LAG3, TIM3, and PD1/PDL1), elevated T regulatory cells, and other immunosuppressor cells, such as tumor-associated myeloid cells, based on previous reports (Lin et al., 2020) (Xiao and Freeman, 2015). One group investigated the prognostic significance of CD274, LAG3, and IDO1 in both tumor cells and infiltrating T cells of MSI-H CRC and found that the expression of these ligands on T cells, and not on tumor cells, was related to a lower risk for recurrence after curative surgery in patients with MSI-H colon cancers. They postulate that an overexpression of these ligands on T cells could lead to adaptive resistance, in which activated T cells trigger a negative feedback mechanism in the tumor microenvironment (Sahin et al., 2019). Primary and acquired resistance to immune checkpoint inhibitors can occur in MSI-H cancers, again best studied in CRC. Though less common, primary resistance to ICI can occur in MSI-H CRCs that lack T-cell infiltration, have a high Treg presence and limited T-cell receptor (TCR) repertoire. Metabolites such as IDO1 and lactate, and suppressive immune cells such as fibroblast infiltration, Treg presence and myeloid-derived suppressor cells are among the extrinsic immune suppression mechanisms (Weber et al., 2018; Sahin et al., 2019; Cui, 2021).

Intrinsic resistance mechanisms may develop from an evolution of tumor genomic instability leading to clonal selection of somatic mutations with survival and oncogenic advantage. A recent study from Grasso et al. (2018) analyzed 1,211 primary CRC samples, including 179 MSI-H tumors, showed significantly mutated genes in immune-modulating pathways and in the antigen-presenting machinery, including biallelic losses of B2M and HLA genes and Wnt/β-catenin signaling genes were significantly mutated in all CRC subtypes, and activation of the Wnt/β-catenin signaling cascade correlated with the absence of T-cell infiltration. Again this showed that the immunoediting processes that MSI-H tumors undergo, which result in genetic events that allow immune escape (Ballhausen et al., 2020). Interestingly, correlation is absent in tumors with B2M mutations, which again supports the important role of MHC downregulation in immune-escape. Though still not well studied in other MSI-H cancers, there has been has also been increasing interest into potential prognostic or predictive biomarkers in MSI-H CRC.

A recent study by Schrock et al. reported significantly higher TMB in responders as compared with non-responders among MSI-H/MMR-D CRC patients who received an immune checkpoint inhibitor–based therapy. Other biomarkers of response which are summarized elsewhere, include but not limited to TMB, CD4/8 T-cell infiltration density, presence/absence of inhibitory myeloid cells in the TME, fibrosis (pertaining to MSI-H colorectal cancers), diversity of TCR repertoire, intact APM/MHC machinery, genomic signatures, microbiome pattern and so forth.

Considering the immunoediting and resistance mechanisms of MSI-H cancers, it is imperative to continue research into combinatory treatments and seek new biomarkers of response and resistance to ICIs. Combination therapies may work by increasing the antigenicity of tumors (with adjuncts such as chemotherapy, radiation therapy or other therapies that might “release” more FSP neoantigen from further DNA damage/mutations, hence increasing the immune response; or targeting immunosuppressive TMEs (Battaglin et al., 2018). Some combination therapies include the use of multikinase inhibtiors, dual inhibitory pathway blockade (anti-PD-1 with CTLA4, anti-PD1 with LAG3, and anti-PD1 with TIGIT), or novel therapies such as JAK1 inhibitors and CSF1R inhibitors (Battaglin et al., 2018).

We foresee combination therapies that may combine chemotherapy, radiotherapy, targeted therapy and immunotherapy (immune checkpoint inhibitors, cancer vaccines and adoptive cell therapy) (Roudko et al., 2021). Multiple combination trials are on-going, including combination of VEGF-inhibitors such as regorafenib and bevacizumab in combination with anti-PD1 therapy. Also, shared neoantigens from frameshift mutations may make off-the-shelf cancer vaccine therapy, whether in the prophylactic setting or in tumor control, a reality for MSI-H cancers (Gebert et al., 2021). This again can be adjunct to the current immune checkpoint inhibitors. On-going work is to use shared neoantigens from common frameshift mutations in the form of preventive or secondary (adjuvant) mRNA cancer vaccine in Lynch Syndrome patients (Hernandez-Sanchez et al., 2022). As mentioned, apart from neoantigen-based therapeutics such as cell therapy or vaccine, other immune checkpoint inhibitors and combination therapies are in various stages of development.

Lastly, the biological drivers in MSI-H cancers with peritoneal disease appear to be distinct from the primary tumors. Understanding of the tumor microenvironment and resistance pattern is paramount in developing novel therapeutic strategies in MSI-H cancers (Ceelen et al., 2020). In addition, the role of the paracrine environment in enclosed biological niches within the peritoneal cavity provides novel avenues for therapeutic perturbation (Gwee et al., 2022). In a recent publication by Hendrikson et al. (2022), ligand inhibition of key drivers of peritoneal carcinomatosis within ascites highlights the possibility of targeting the fluid microenvironment. Exploiting the biological drivers in MSI-H cancers within the cancer cells and microenvironment is highly attractive and further translational work and therapeutic trials in this area is anticipated.

## Conclusion

In the era of precision genomic profiling and novel immunotherapeutics, MSI-H cancers have become increasingly targetable. We summarized in this review clinical, genomic, tumor-immune landscape including paracrine dependence, treatment considerations for MSI-H cancers, and see patterns of convergence and divergence in this unique group of cancers. The treatment paradigm will continue to evolve with increased understanding of microsatellite-instability on the tumor and its underlying immunobiology. Future combination therapies may improve the response rate and outcomes further in this group of genomically unstable, highly immune-cell-rich tumors. We also note peritoneal metastases to be an important consideration in recurrent/metastatic MSI-H colorectal and gastric cancers.
